# Cannabis industry campaign expenditures in Colorado, 2005–2021

**DOI:** 10.1016/j.drugpo.2023.104156

**Published:** 2023-08-07

**Authors:** Thomas Rotering, Stella Bialous, Dorie Apollonio

**Affiliations:** a Department of Clinical Pharmacy, University of California. UCSF Clinical Sciences Box 0622, 521 Parnassus Avenue, Floor 3 Room 3303, San Francisco, CA 94143, USA; b School of Nursing, University of California. 490 Illinois St., Floor 12, Box 0612 San Francisco, CA 94143, USA

**Keywords:** Cannabis, Public Health, Colorado, Politics, Conflict of Interest

## Abstract

**Background:**

The cannabis industry has been described using the commercial determinants of health framework because it seeks to increase sales and profits through efforts to change the political environment. To increase understanding of these cannabis industry’s efforts, this study describes cannabis industry campaign contributions in Colorado through an analysis of public records.

**Methods:**

We reviewed datasets posted online at the Colorado Secretary of State’s Transparent in Contribution and Expenditure Reporting (TRACER) Campaign Finance System. We generated descriptive data on cannabis industry contributions to elections and conducted regressions to identify predictors of contributions, and associations between lagged contributions and a legislator’s cannabis industry score (how closely aligned lawmaker’s legislative history is with the cannabis industry from −1 to 1).

**Results:**

Between 2005–2021, 429 cannabis-affiliated contributors gave $4,658,385 (2021 inflation-adjusted) to 512 electoral committees. Contributions came primarily from non-profits (45%), businesses (27%), and individuals (25%). After recreational legalization in 2012, contributions from non-profit donors with industry ties gave way to contributions from cannabis businesses, business owners, and lobbyists. Cannabis industry contributions to local and state-wide ballot initiative campaigns historically made up the majority of the industry spending, but contributions to candidate committees more than tripled between the 2009–2010 legislative cycle and the 2019–2020 cycle. From 2017–2020, every $10,000 in lagged campaign donations from cannabis industry affiliated contributors was associated with a 0.245-point increase in a legislator’s cannabis industry score (p=0.04).

**Conclusion:**

Cannabis-affiliated interests made substantial campaign contributions in Colorado. Public health advocates should monitor industry connections to lawmakers and industry tactics used to mask the source of political contributions. Continued surveillance of the cannabis industry is essential to exposing conflicts of interest and preventing undue industry influence.

## Background

As of December 2022, recreational cannabis was legal in Canada, Malta, Uruguay, and 21 US states, two US territories, and the District of Columbia, and was poised to reach a market size of $198 billion globally by 2028 (BBC News, 2022; Hansen, Alas, & Davis, 2022; Fortune Business Insights, 2021). Research has found that cannabis use increases in states that have legalized recreational cannabis, though higher use in these states before legalization complicates the explanation (Parnes et al., 2018; Jeffers et al., 2021; Stolzenberg et al., 2016; Wall et al., 2016; Zellers et al., 2023).

Cannabis-derived and synthetic cannabis-related drug products, including cannabidiol, dronabinol, and nabilone, are approved by the FDA for treatment of select conditions such as certain seizures and for therapeutic uses, including for nausea associated with cancer chemotherapy and for the treatment of anorexia associated with weight loss in AIDS patients (Food and Drug Administration, 2023). However, among adults who use cannabis for either medical or recreational reasons, smoking for recreational purposes is the most commonly reported method of administration (Dai & Richter, 2019; Steigerwald, Wong, & Cohen, 2016). Although recreational cannabis use is often perceived to be harmless, cannabis smoke contains many, and in some cases more, of the same chemicals found in cigarette smoke (Ott et al., 2021; Moir et al., 2008). Cannabis use can also lead to lung damage, ischemic stroke, aneurysm, more frequent chronic bronchitis episodes, and heart attack, and cardiovascular disease (Hancox et al., 2021; National Academies of Sciences, 2017; Rumalla, Reddy, & Mittal, 2016a, 2016b; Shah et al., 2020). The addictive potential of cannabis is comparable to that of opioids, and use is especially risky for those who use cannabis frequently or begin use at an early age (Hasin et al., 2015; Lopez--Quintero et al. 2011; National Institute of Drug Abuse (NIDA), 2021; National Institute of Drug Abuse, 2020; Winters & Lee, 2008). Cannabis use is disproportionately concentrated in young, medically underserved populations, including Blacks and Native Americans, as well as those reporting less educational attainment (Jeffers et al., 2021).

The World Health Organization defines commercial determinants of health as the “private sector activities that affect people’s health, directly or indirectly, positively or negatively (World Health Organization, 2023). Legalization has placed the cannabis industry in the private sector and, in light of the detrimental effects of cannabis use on health, (Hancox et al., 2021; National Academies of Sciences, 2017; Rumalla, Reddy, & Mittal, 2016a, 2016b; Shah et al., 2020) the cannabis industry has been described using the commercial determinants of health framework; (Kickbusch, Allen, & Franz, 2016; Adams, Rychert, & Wilkins 2021a; Adams, Rychert, & Wilkins, 2021b) previous research has noted that the cannabis industry seeks to increase sales and profits by making efforts to change the political environment and alter public perception through corporate social responsibility programs (Wakefield, Glantz, & Apollonio, 2022). Cannabis industry interests may use political means to push for profitable free market models of cannabis legalization that favor vertical integration (i.e., ownership and operation of every part of the supply chain), large scale commercialization, and consolidation over models like the state-sponsored monopoly model which do not (Room & Jenny, 2019). Research on commercial determinants of health has explored the influence that the tobacco, alcohol, pharmaceutical, and food industries exert on policy, but research on cannabis is limited (Mialon, 2020). Campaign contributions from alcohol, tobacco, pharmaceutical, and food interests have been found to create a sense of reciprocity with elected representatives (Gostin, 2016; Open Secrets, 2021; Luke and Krauss, 2004; Robbins, 2018; Wouters, 2020; OECD, 2017). Alternatively, companies may “invest” in candidates who are already aligned with the interested party to support their policy goals, rather than to buy influence (Goldberg et al., 2020). The tobacco industry has also campaigned on ballot initiatives, proposed laws or referendums that voters consider on the ballot, to create favorable environments for their products (Fallin, Grana, & Glantz, 2023; Goel et al., 2021; Laposata, Kennedy, & Glantz, 2014; Sircar & Glantz, 2021). Cannabis industry political contributions are likely motivated by similar policy objectives. Moreover, tobacco and alcohol companies have invested in cannabis companies and maintain formal lobbying partnerships with them, increasing the likelihood that similar tactics will be employed across industries as strategic partnerships mature (Roberts, 2021; Auxly Cannabis Group Inc., 2019; Sabghai, 2021; Nair, 2020; CPEAR, 2020; Barry, Hilamo, & Glantz, 2014).

Although some peer-reviewed research and journalism has examined cannabis industry influence on decisions to legalize cannabis, and news reports have periodically characterized cannabis industry political contributions, peer-reviewed literature on the cannabis industry’s efforts to influence politics after legalization is limited (Orenstein and Glantz, 2020; Graham 2017; Roberts, 2020; Gunelius, 2020; Gangitano, 2019; Cadelgado, & Miller 2016; Ingraham 2017; Gurciullo, 2015; Ramstack, 2019). Observers have suggested that funding for cannabis legalization measures and pro-cannabis candidates will come less from philanthropists and non-profit associations (e.g., the Marijuana Policy Project and the Drug Policy Alliance) and more from trade groups and multi-state organizations as the industry develops (Schroyer, 2020; Akin, 2019). In states like Colorado, where legal cannabis sales exceeded $2.2 billion in 2021, the cannabis industry has the legal standing, resources, and incentives to expand markets and advance regulatory environments favorable to growth (Colorado Department of Revenue, 2022). To increase understanding of the cannabis industry’s efforts to influence politics, this study describes cannabis industry influence on electoral politics over time in Colorado through an analysis of public records. By their nature, these kinds of contributions are intended to influence public policy, either by changing the makeup of a legislature so that individuals that support contributors’ issues take or hold office, by directly influencing elected representatives to vote in favor of preferred positions, or both. Colorado was the first state in the US to legalize recreational cannabis in 2012, making it possible to track changes in political contributions over a decade and assess whether predictions that the cannabis industry would transition to operating like other industries were validated (Hansen, Alas, & Davis, 2022). We anticipated that like other industries (Mialon, 2020; Gostin, 2016; Luke and Krauss, 2004; Robbins, 2018; Wouters, 2020; OECD, 2017; Goldberg et al., 2020; Sircar & Glantz, 2021; Laposata, Kennedy, & Glantz, 2014; Goel et al., 2021), cannabis companies would increase monetary spending on political contributions over time, leverage multi-state networks to promote candidates and policies favorable to their growth, and oppose taxes and regulation. We also expected that candidates who received more funding from cannabis affiliates voted in ways consistent with stated positions of cannabis companies and trade associations.

## Methods

This retrospective study reviewed datasets that were posted online at the Colorado Secretary of State’s Transparent in Contribution and Expenditure Reporting (TRACER) Campaign Finance System to describe cannabis industry contributions between December 1, 2004 (the beginning of the 2005 campaign and filing calendar start date) and November 30, 2021 (the end of the 2021 post-election reporting period). These were combined with public information on voting records of state legislators, gathered from the Colorado General Assembly’s website (Colorado General Assembly, 2022). The review was conducted between June 2021 and March 2022.

### Setting and data

Campaign contributions: Colorado election law requires candidates and committees to register and disclose financial information to the Secretary of State in scheduled intervals via TRACER, which allows anyone to export information on candidates, committees, contributions, and expenditures (Colorado Secretary of State, 2021). Contribution details, electioneering disclosures, and major contributor reports are filed by a committee’s registered agent. Between June 2021 and February 2022, we compiled a database of disclosed cannabis industry activity in electoral politics using TRACER.

Affiliations: To identify cannabis industry contributions, we first searched the TRACER database by contributor using a list of known cannabis industry businesses, trade associations, and affiliated individuals identified in an earlier study of cannabis industry lobbying in the Colorado state legislature (Rotering & Apollonio, 2022). The resulting list of cannabis industry contributions was expanded upon through a review of the recipient committees to which cannabis businesses donated. We identified additional contributors as cannabis industry business interests if they a) were organizations or individuals that held a cannabis business license (e.g., licensed retail or medical store, cultivation facility, manufacturer, testing facility, or transporter), b) were organizations that shared board members, owners, or investors with a cannabis company, c) were organizations that disclosed members that were cannabis businesses, or d) were organizations or individuals who would directly profit from cannabis sector growth (e.g., pharmaceutical companies that sell cannabis derived drugs, cannabis focused consultants, investors, lab services, or employee training services). Individuals and companies were identified as affiliated with cannabis interests by cross-referencing names and addresses with the Colorado Department of Revenue MED Verification Tool and the Secretary of State’s online business Database Search Tool (Colorado Secretary of State, 2022a, Colorado Secretary of State, 2022b). From this updated list of contributors, we also added information on contributions from additional cannabis industry affiliated individuals when the TRACER entry listed their employer as a cannabis business or as a consultancy that retained cannabis industry clients. For a complete list of inclusion criteria defining cannabis industry affiliated organizations and individuals, see Supplement, Table 1.

We continued the snowball search by querying TRACER for all contributions from the expanded list of cannabis industry businesses, trade associations, and affiliated individuals. We then standardized contributor names after verifying identity using the address, employer, and occupation fields and conducting supplementary internet searches; details of this process were described in our dataset using a text field. We generated 4,095 observations with 15 variables.

For each TRACER entry identified as being from a cannabis industry interest, we noted the contributor name, whether the contribution was on behalf of an individual or an organization (and type of organization), nature of the link to the cannabis industry, location, the date and amount of payment, the recipient committee’s name and committee type (issue committee, candidate committee, etc.; see Supplement, Table 2), and whether the contribution was in-kind, or a major contribution (a contribution over $1,000). Some campaigns reach the end of an election cycle with excess funds that may be returned to the contributor; these are noted as a negative dollar amount and were included to reflect the final total. We adjusted payment amounts for inflation in November 2021 dollars using consumer price index (CPI) data from the U.S. Bureau of Labor and Statistics (Bureau of Labor and Statistics, 2022).

Voting scores: To examine the connection between cannabis industry political contributions and the voting records of the lawmakers to which they donate, we compared the legislative voting records for all members of the two most recent (71st (2017–2018) and 72nd (2019–2020)) Colorado General Assemblies with the official lobbying positions of the Marijuana Industry Group (MIG), the largest and most influential cannabis industry trade association in Colorado. Legislative voting records were gathered from the Colorado General Assembly’s website and we queried the Secretary of State’s Client Activity Search Tool for data on official MIG lobbying positions (Colorado General Assembly, 2022; Colorado Secretary of State, 2022). The MIG lobbied to support or oppose 18 bills in this time frame. We excluded bills that the MIG only monitored or lobbied to amend at any point because it was not clear if legislators voted in line with industry interests for those bills. The floor and committee votes for each legislator were assigned a value of −1 if opposite the MIG position and +1 if aligned with MIG. The sum of these votes by session was then divided by the total number of voting opportunities to establish a cannabis industry score ranging from −1 to 1.

Legislator characteristics: We also gathered information on party, district, whether the session was the legislator’s first, if they held leadership positions, and if so what those positions were, and how much money each legislator’s committee received from the cannabis industry either through candidate committee contributions or by benefiting from independent expenditures committees, political committees, or small donor committees, where they could be linked to a specific candidate through information on the TRACER committee profile.

### Measures

Our primary outcome measure was contribution amounts. We also considered variables indicating the nature of the cannabis industry relationship, date of contribution, contribution type and location, recipient, and recipient committee type. Our secondary outcome measure was the coded legislative voting record for members of the Colorado General Assembly from 2017–2020 on cannabis bills that MIG either supported or opposed.

### Analytical strategy

We generated descriptive data on cannabis industry contributions to Colorado elections over time and conducted subgroup analyses to determine the proportion of contributions from individuals and contributions originating out-of-state. We then modeled predictors of cannabis industry contributions using legislator characteristics, and associations between cannabis industry score and lagged contributions, using ordinary least squares (OLS) regression for two election cycles (2017–2018 and 2019–2020). We chose to limit our analysis to these two cycles because 1) the 2021–2022 election cycle had not yet finished and so contributions data was not yet complete, 2) our goal was to present a snapshot of the association between contributions and voting behavior post-legalization rather than a historical trend, 3) these two most recent cycles included a high number of both recreational and medical cannabis related bills than prior years, allowing for better measures of association, 4) these two most recent reported cycles included the first and second greatest amount of cannabis industry contributions to candidates in an election cycle, and 5) given that Colorado has a term-limited legislature, these two election cycles included information on legislators and cannabis industry affiliated entities that were most likely to still be relevant. All statistical analysis was completed using Stata v17 (Long & Jeremy, 2014).

Rather than describing each ballot issue that the cannabis industry contributed to, we present a demonstrative example of how the cannabis industry uses campaign funding to oppose regulation, we also present a case study of the campaign for ballot initiative 139 (2016). This case study triangulates data from multiple of the above publicly available sources to describe and give context the financial power, legal tactics, and message frames employed by the industry.

## Results

In the 2005–2021 campaign cycles, 429 cannabis industry affiliated contributors gave $4,658,385 (inflation-adjusted to 2021) to 512 different recipients. Contributions came from non-profits (45%), businesses (27%), individuals (25%), trade associations (3%), and labor unions (<1%).

### Increasing contributions over time

Overall, non-profits gave 45% ($2,110,801) of tracked contributions. Before recreational cannabis legalization in November of 2012 by Proposition 64, political campaign contributions favorable to the cannabis industry were made primarily by non-profit groups seeking to legalize cannabis and based in Washington D.C. Contributions from non-profits in the 2005–2014 campaign cycles composed 83% of total contributions, but only composed 1% of those contributions in the 2015–2021 cycles.

The Marijuana Policy Project (MPP) and its associated foundation gave $1,802,442 (85% of all non-profit contributions) to efforts to legalize and implement policies for recreational marijuana. MPP has industry ties. Rob Kampia, MPP founder, CEO (2000–2008), and secretary of the Board of Directors (2009–2017) for MPP, also founded the National Cannabis Industry Association, a cannabis industry lobbying group. Chris Woods, owner of the cannabis company Terrapin Care Center, sat on the board of MPP from 2016–2017. Mason Tvert served as director of communications for MPP and co-founded a prominent committee supporting recreational cannabis legalization (SAFER Colorado) before his current roles as communications advisor at the cannabis industry law firm Vicente Sederberg LLP and a partner at the VS strategies, the law firm’s public affairs affiliate (Vicente Sederberg LLP, 2022). Neal Levine, former director of state campaigns for MPP from 2003–2009, was Senior Vice President of Government Affairs for LivWell Enlightened Health from 2015–2018 before serving as director of the Cannabis Trade Federation from 2018–2020. Justin Hartfield of Weedmaps also donated to MPP campaigns and served on the board of directors in 2012 (Marijuana Policy Project, 2012). Patrick McManamon of Cannasure, a cannabis-focused insurance company, has also donated to MPP campaigns (Summers 2018; Cannabis Business Times 2018).

After the 2013–2014 cycle, contributors other than non-profits, including lobbyists representing the cannabis industry, businesses, individuals, and trade associations with ties to the industry made nearly all contributions.

Trade associations gave 3% ($126,766) of tracked contributions, primarily to independent expenditures committees, issue committees, and 527 Political Organizations. The Marijuana Industry Group (formerly the Medical Marijuana Industry Group) gave $69,965 (representing 55% of contributions from trade associations). Colorado Leads gave $44,229 (35% of contributions from trade associations). These trade associations listed several of the same cannabis companies as members (e.g., LivWell, Native Roots, and Lit) and both organizations also lobbied the state legislature.

Between December 1, 2004, and November 30, 2021, cannabis businesses gave 27% ($1,243,654) of tracked contributions. Beyond Broadway, a vertically integrated multi-state cannabis retailer doing business as Livwell, was the top business contributor at $274,072 (22% of business contributions). Business contributions peaked in the 2015–2016 cycle at $738,489.

Cannabis industry-affiliated individuals gave 25% ($1,162,167) of tracked contributions from 2006–2021. Cannabis industry affiliated individuals include employees and consultants of cannabis businesses, though the largest contributions were made by C-suite executives or owners of cannabis businesses. For example, Christopher Woods, owner of The Genetic Locker, a vertically integrated multi-state cannabis retailer doing business as Terrapin Care Station, spent more than any other individual at $89,865 inflation-adjusted (8% of individual contributions). Some contributions we identified as being made by individuals who owned a cannabis business listed themselves as self-employed, without any indication of a cannabis affiliation (for a list of the top 15 cannabis industry affiliated contributors, see the Supplement, Table 3).

### Multi-state networks of contributors

Out-of-state sources comprised 41% ($1,913,834) of contributions while in-state sources comprised 59% ($2,744,551). Contributions from the Washington D.C. based MPP and its foundation were 94% of all out-of-state contributions. Ninety-six percent of contributions from California ($34,162) came after California legalized recreational cannabis in November of 2016 and were made primarily by Eaze solutions, a cannabis delivery company, and Justin Hartfield, former CEO of WeedMaps and board member of both NORML and MPP.

### Promotion of candidates and policies

Contributions from the cannabis industry and affiliates were made to multiple types of recipients. The majority were dedicated to issue committees (66%) that supported or opposed ballot initiatives (Colorado Secretary of State, 2021). Candidate committees, which differ from issue committees because they can only accept contributions and make expenditures on behalf of a candidate, received 14% of contributions (Colorado Secretary of State, 2021). Independent expenditure committees, which seek to oppose or support a candidate without coordinating with any candidate, received 9% of cannabis industry contributions (Colorado Secretary of State, 2021). Tax-exempt federally regulated political organizations known as 527 political committees received 6% of contributions. Political committees (which support or oppose the nomination or election of one or more candidates) received 3%, political parties (groups of registered electors who nominate candidates for office) received 3%, and small donor committees (political committees that accept donations of $50 or less per person, per year) received 0.04% (see the Supplement, Table 2) (Colorado Secretary of State, 2021). Contributions to ballot initiative committees, primarily those proposing recreational legalization, dominated early spending, but contributions to candidate committees increased steadily over time from $30,795 in the 2009–2010 cycle to $185,223 in the 2018–2019 cycle.

Among contributions to committees that listed a political affiliation, 77% ($1,181,580) went to Democrats, 20% (314,871) went to Republicans and 3% ($38,654) went to other parties or those who listed their political affiliation as non-partisan. Recipients included Attorney General Phil Weiser (Democratic Party, $35,960), Secretary of State Jena Griswold (Democratic Party, $21,457), and representative Dan Pabon (Democrat, $18,762), who served in the Colorado House of Representatives for 8 years, holding the position of State House Majority Leader from 2013–2014 before becoming a Vice President at Sewald Hanfling Public Affairs in 2018 and then general counsel for Schwazze, a cannabis company, in 2019 (for a list of the top 15 recipients of cannabis industry funds, see the Supplement, Table 4).

### Associations between industry funding and legislative voting records (scorecards)

We found a statistically significant association between the amount of inflation adjusted campaign donations that a legislator received from the cannabis industry prior to the legislative session, and the degree to which their votes aligned with the lobbying position of the Marijuana Industry Group in that session (see Table 1). Specifically, every $10,000 in campaign donations received prior to the 2017–2018 cycle (lagged contributions) was associated with a 0.457-point increase in a legislator’s cannabis industry score, which ranged from −1 to 1, in the 2017–2018 cycle (p < 0.01). The increase in score was larger for inflation-adjusted campaign donations from consultants who had cannabis industry clients than it was for donations from all sources (which included business and individuals who were not industry consultants). Every $10,000 in lagged contributions from consultants who had cannabis clients was associated with a 1.432-point increase in a legislator’s cannabis industry score (p<0.01) in the 2017–2018 cycle. Results are provided in Table 1.

### Predictors of contributions

We also considered whether the predictors of cannabis contributions were consistent with our descriptive findings, which suggested higher spending on Democratic candidates. In regressions reviewing the associations between cannabis contributions in the 2017–2018 and 2019–2020 election cycles and legislative body, leadership positions, time in office, and political party, Republicans received significantly smaller contributions than Democrats in 2017–2018 ($668 less). In the 2017–2018 cycle, legislators in the state Senate received significantly smaller cannabis contributions than legislators in the state House ($812 less). However, leadership status and time in office did not appear to be associated with the level of cannabis contributions. Results are provided in Table 2.

### Opposition to taxes and regulation case study: The Colorado health research council

In 2016, the Colorado Health Research Council (CHRC) raised $592,788 but spent only $197,331 to oppose statewide ballot initiative 139 (I-139), a measure which would have required retail cannabis sold in the state to be 1) in child-resistant packaging, 2) in individually packaged, single serving units, 3) labeled with the health risks and potency of packaging, and 4) limited to a potency of 16% tetrahydrocannabinol (THC) (Colorado Secretary of State, 2022). Ninety-six percent of contributions to the CHRC were from industry affiliated sources including cannabis dispensaries, manufacturers, growers, and business owners. The largest contributions all came from cannabis businesses and affiliates, including a $97,208 in-kind contribution of campaign staffers from Beyond Broadway LLC, two checks worth $140,656 from The Genetic Locker INC, and $25,000 checks from Choice Organics INC, Dylan Consulting Company, Futurevision LTD, Left Bank LLC, Native Roots, Renaissance Solutions INC, and RK Enterprises LTD. The TRACER committee detail page for the CHRC lists Dean Heizer, the Executive Director and Chief Legal Strategist for Livwell (a trade name of Beyond Broadway LLC), as the registered agent.

The Healthy Colorado Coalition was the main proponent of I-139 and spent a total of $3,258 (on printing services) to support the initiative. The address listed in the TRACER website is for a townhouse in Boulder, Colorado. Frank McNulty, the former Colorado Speaker of the House, represented the coalition as their attorney (2016).

The initiative’s title was first set on April 21, 2016, and the cannabis industry immediately petitioned the Colorado Ballot Title Setting Board to hear complaints that the initiative was unclear and misleading and that it impermissibly addressed multiple subjects (Seman & Paul, 2016a; Colorado Secretary of State, 2016). The movants of this petition included Dean Heizer and Gregory Kane, with counsel from Heizer Paul LLP, a cannabis industry affiliated law firm, and JPS Law Group. The title board denied the motion for rehearing on April 29. However, on May 6, the petitioners filed an appeal with the Colorado Supreme Court (Seman & Paul, 2016b). According to TRACER expenditure reports, CHRC paid both Heizer Paul LLP ($10,491) and JPS Law Group ($30, 000) for “Consultant and Professional Services” during the summer of 2016. The case was not resolved until June 16, 2016, when the Colorado Supreme Court awarded the Healthy Colorado Coalition the victory and affirmed the title board’s motion to deny a rehearing of the ballot title (Colorado Supreme Court, 2016).

With the legal challenges resolved, advocates for I-139 had less than two months left to collect 98,492 signatures by August 8, 2016, which were needed for it to appear on the ballot (McKibbin, 2016). In July 2016, Frank McNulty told the Gazette that an anti-139 consultant shared that the anti-139 effort paid ballot signature collecting firms, “…$75, 000 to $200,0000, depending on size of each company, to get contracts that say they will not gather signatures for this ballot measure” (McKibbin, 2016). McNulty also told the Denver Post that the anti-139 campaign had paid a Colorado Springs signature collection firm, Kennedy Enterprises, up to $200,000 to refuse to collect signatures for them (Denver Post Editorial Post, 2016). However, these accusations were denied by Neal Levine of CHRC, who at the time was the senior vice president of government affairs for LivWell (2016). Jo McGuire, a Healthy Colorado Coalition consultant, also claimed that one out-of-state firm had refused to work with them because “they had signed a no-compete agreement that prevented them from working with us” (McKibbin, 2016). Tyler Henson, who at the time was president of the Colorado Cannabis Chamber of Commerce, denied payments to signature firms (McKibbin, 2016). Expenditures reports filed by the CHRC available through TRACER show payments to PAC/WEST ($110, 620) and RBI Strategies and Research ($27,300) for consultant and professional services but did not list payments to Kennedy Enterprises or signature collecting firms.

I-139 was withdrawn on July 8, 2016. Mike Elliot, executive director of the Marijuana Industry Group, observed that “[I-139] would gut Amendment 64,” banning most cannabis products because they contain over 16% THC (Scroyer, 2016). Ali Pruitt, a representative and the registered agent for the Healthy Colorado Coalition said in a press release that “…we simply couldn’t keep up with the financial costs brought on by the underhanded and baseless delays used by the marijuana industry to keep us off the ballot. The marijuana industry built a wall of money… that we couldn’t break through” (Healthy Colorado Coalition, 2016). With I-139 defeated, the CHRC returned $298,248 to contributors in August of 2016.

## Discussion

Non-profit organizations, businesses, trade associations, and individuals affiliated with the cannabis industry dedicated over $4 million in 2021 dollars to campaign contributions between the 2005 and 2021 campaign cycles. While contributions from non-profit organizations based in Washington D.C. dominated early industry spending prior to recreational legalization in 2012, donations after legalization came primarily from cannabis businesses, business owners, and consultants. Cannabis industry contributions to local and state-wide ballot initiative campaigns historically made up the majority of industry spending, but contributions to candidate committees more than tripled between the 2009–2010 legislative cycle and the 2019–2020 cycle. Increasing contributions from cannabis business interests to candidate committees may indicate growing interest in securing political influence.

Candidates who received campaign contributions from the cannabis industry were statistically more likely to vote with the cannabis industry in the 2017–2018 election cycle, as measured by alignment with the lobbying positions of the Marijuana Industry Group. This may be because cannabis interests contributed to candidate campaigns to buy influence, or because they were investing in candidates who already hold favorable views towards the cannabis industry. Campaign contributions may also be a part of a larger strategy to secure access to legislators, which includes lobbying activities and corporate social responsibility programs (Apollonio, Cain, & Drutman, 2008; Mayer and Mujumdar, 2014; Wakefield, Glantz, & Apollonio, 2022). This relationship was larger for contributions made by consultants with cannabis industry clients. These industry operatives may be able to convert political contributions into influence more effectively than the broader industry ecosystem because of closer personal or professional relationships which extend into the legislative session in the form of lobbying (Rotering & Apollonio, 2022). Alternatively, consultants may be able to better target contributions to those legislators who hold more favorable views towards the industry because they have a more accurate understanding of legislator views. The association did not hold for the 2019–2020 election cycle, which may be explained by the smaller number of cannabis-related bills in the 2019–2020 election cycle, their limited scope, or changing legislative priorities (e.g., COVID-19).

Given the multiple businesses, business owners, consultants, and affiliated organizations representing the cannabis industry, transparency in campaign finance reporting is important for monitoring their activities. We found that campaign finance records for organizations and individuals often lacked disclosure of their cannabis affiliation, making it difficult to determine whether a contribution was industry-affiliated without conducting substantial additional research. These tactics of obfuscation have also been employed by the tobacco and alcohol industries to manipulate public perception and advance industry-favorable policies (Apollonio & Bero, 2007; Laposata, Kennedy, & Glantz, 2014; Luke and Krauss, 2004; Savell, Fooks & Gilmore, 2016). Recommendations made based on this research could be applicable to the cannabis industry, including efforts to exclude corporate actors from policymaking, careful surveillance, strong government regulation, or public monopolization. To address lack of transparency, the Colorado Secretary of State could mandate that recipient committees include a cannabis affiliation in the employer, occupation, or occupation comments fields of the campaign finance records available in the TRACER system. For committees that are managed and run by cannabis businesses and their affiliates (such as the Colorado Health Research Council), the TRACER committee detail page could include a disclosure that the committee is affiliated with the cannabis industry.

Industry-led ballot initiative committees like the Colorado Health Research Council demonstrate the disproportionate financial resources that cannabis interests can apply to counter grassroots efforts to regulate cannabis. These financial resources were in part directed towards law firms that used litigation to obstruct the placement of ballot initiative 139. Legal challenges to I-139 brought under the single-subject rule have also been employed by the tobacco industry to unsuccessfully challenge tobacco tax propositions 99 (1991) and 10 (2003) in California (Huynh and Huynh, 2016).

### Strengths and limitations

Our study has limitations. We identified cannabis affiliations that were not explicitly listed in contribution reports by searching public information sources, and it is likely that not every cannabis interest was identified. Some contribution reports were missing employer, address, or occupation information and would list a cannabis affiliation when donating to certain committees but not when donating to others. We addressed this by including all contributions from contributors whose identity could be verified using name and address. Cannabis businesses may make anonymous donations through companies with which they share an owner, law firms, and/or public relations agencies that do not disclose a cannabis affiliation, meaning the total contributions reported in this study may be an undercount. We attempted to identify these contributions by cross-referencing the names of owners, board members, and founders with contribution data to determine if a company held a cannabis affiliation, however it is unlikely that every contribution was identified, and it is possible that companies with the same owners have different interests. Our inclusion criteria could also be overly broad, incorporating contributions from hemp and pharmaceutical industry donors whose interests may be substantial different from the broader cannabis industry, even though they often participate in cannabis trade association groups. To account for these possibilities, we conducted subgroup analyses of public relations firms and different donor classes, making our analysis the most detailed and current assessment of cannabis industry influence after legalization that we have identified. This retrospective study was observational in nature, and we cannot assess causality.

Future research should make efforts to determine the direction of causality when considering the associations between campaign contributions and voting records. Qualitative research involving key informant interviews and documentary research aided by Freedom of Information Act requests could further characterize the ways in which cannabis industry involvement in electoral politics, including lobbying, interacts with downstream policy goals. Similar research should be conducted in other states and the federal government to characterize cannabis industry political activity in other contexts.

## Conclusion

**Fig. 1.**
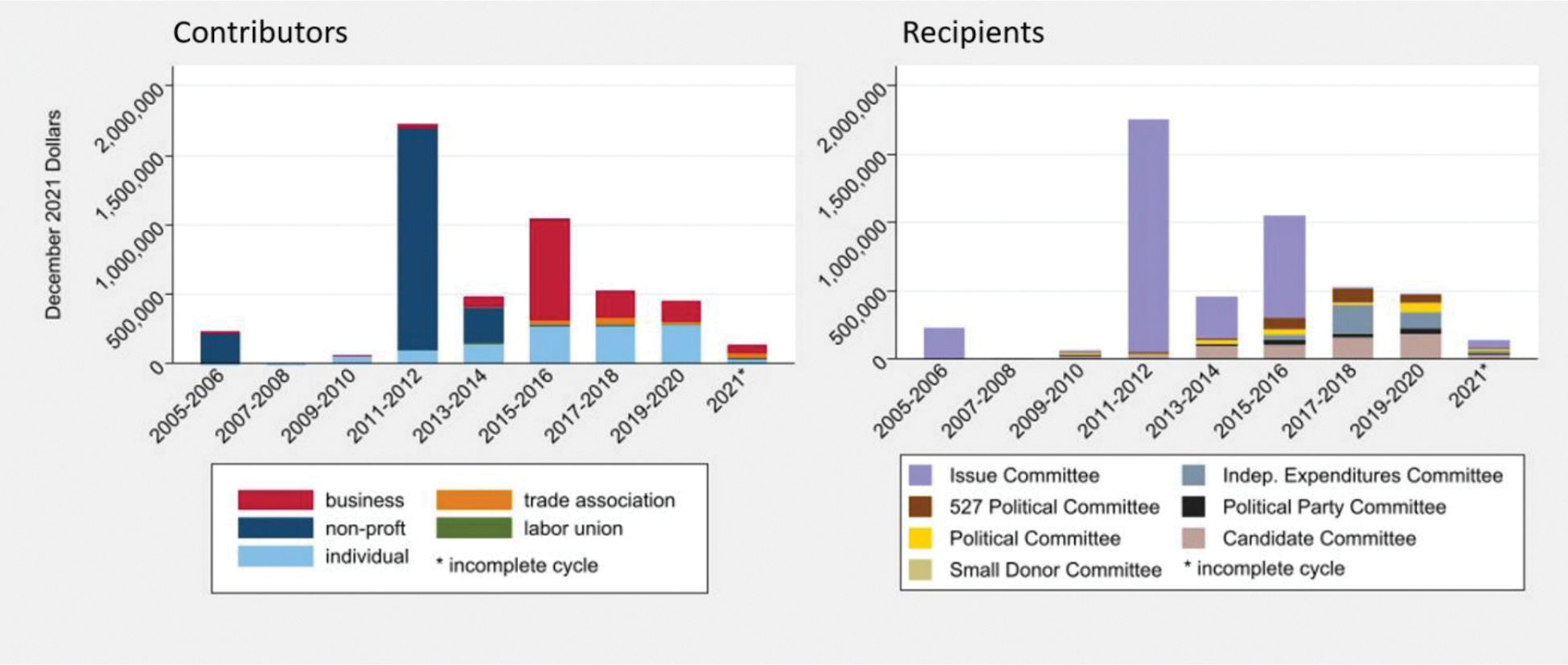
Cannabis contributions over time by contributor and recipient type

**Fig. 2.**
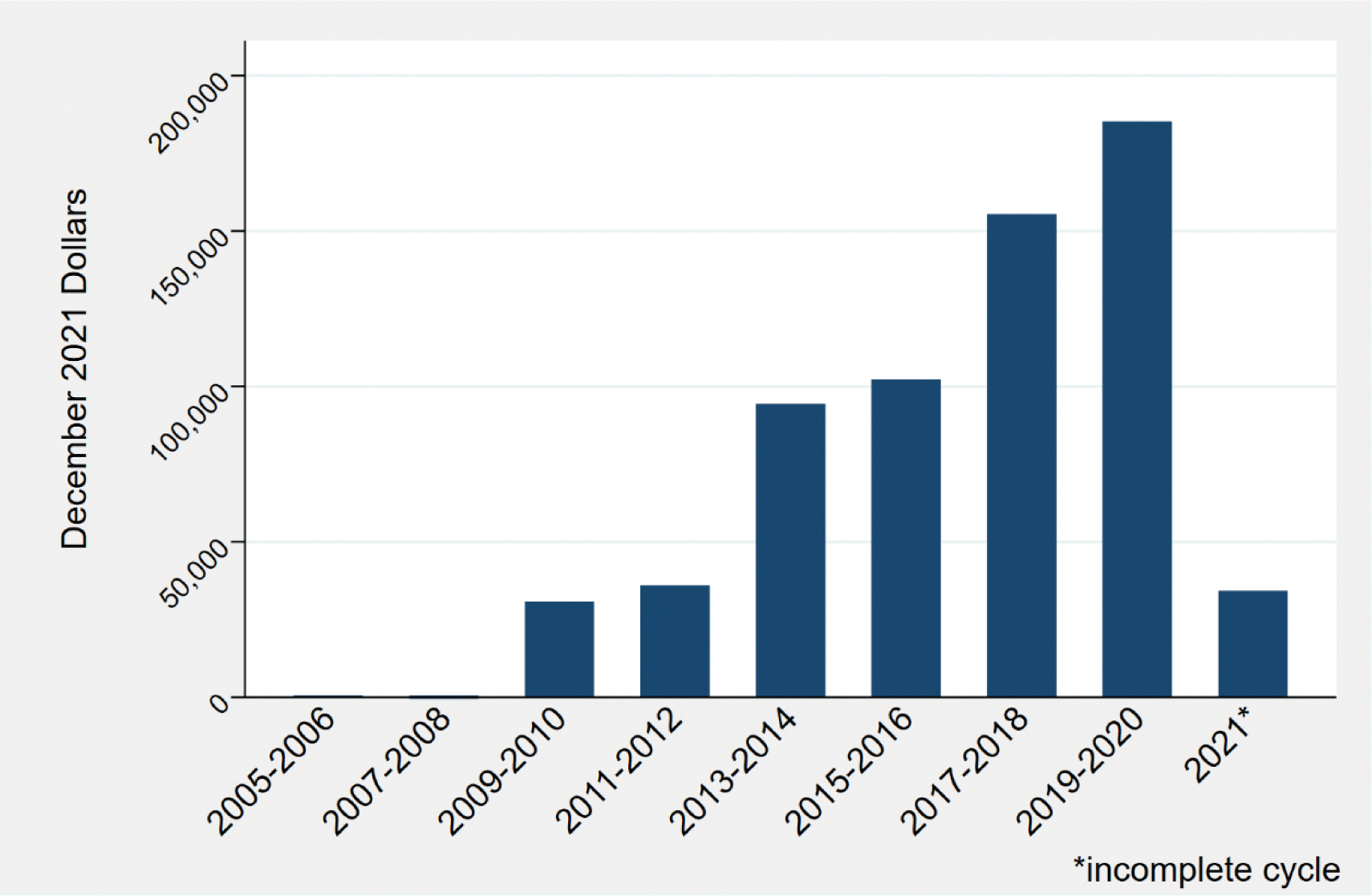
Cannabis industry political contributions to candidate committees 2005–2021

Like other industries implicated as commercial determinants of health, the cannabis industry, as well as organizations and individuals linked to it, made substantial campaign contributions in Colorado both before and after cannabis legalization. By their nature, these kinds of contributions are intended to influence public policy, either by changing the makeup of a legislature so that individuals that support contributors’ issues take or hold office, by directly influencing elected representatives to vote in favor of preferred positions, or both. Public health advocates should be aware of the extensive political connections between the industry and lawmakers as well as industry tactics used to mask the source of political contributions, and governments should improve reporting to increase transparency. Continued surveillance of the cannabis industry is essential to expose hidden conflicts of interest and prevent undue industry influence Fig. 1, Fig. 2.

## Supplementary Material

supplement

Supplementary material associated with this article can be found, in the online version, at doi:10.1016/j.drugpo.2023.104156.

## Figures and Tables

**Table 1:** Associations between voting score and lagged contributions by election cycle 2017–2020 (in $10,000s)

Association (coefficient ± SE)	2017–2018		2019–2020		Overall	

All sources	0.457 ± 0.166	P < 0.01**	.0804 ± .156	p = 0.61	0.245 ±0.117	P = 0.04*
Consultants only	1.432 ±0.506	p < 0.01**	0.428 ± 0.432	p = 0.32	0.800 ±0.339	P = 0.02*
Other contributors	0.551 ± 0.225	**P = 0.02**	0.053 ± 0.230	p = 0.82	0.296 ±0.166	p = 0.08

**Table 2:** Predictors of contributions made by cannabis interests in the 2017–2018 and 2019–2020 election cycles

Predictors (coefficient ± SE)	2017–2018		2019–2020		Overall	

Legislative body						
• House	reference	reference	reference	reference	reference	reference
• Senate	−812.1 ±343.3	p = .02*	−1430.8 ±1266.0	p = 0.26	−1009.3 ±652.3	p = 0.12
Leadership status						
• None	reference	reference	reference	reference	reference	reference
• Holds leadership position	304.5 ±404.8	p = 0.45	−708.5 ±1448.0	p = 0.63	−170.8 ±756.8	p = 0.82
Time in office						
• More than 1 term	reference	reference	reference	reference	reference	reference
• First term	−269.6 ±379.0	p = 0.48	−1534.0 ±1249.5	p = 0.22	−770.0 ±659.3	p = 0.24
Political party						
• Democrat	reference	reference	reference	reference	reference	reference
• Republican	−668.3 ±305.9	p = 0.03*	−1710.4 ±1128.7	p = 0.13	−1265.1 ±582.2	p = 0.03*

## Data Availability

The data supporting the conclusions of this article are publicly available at https://tracer.sos.colorado.gov.
