# Supplementary material for: Cannabis industry campaign expenditures in Colorado, 2005–2021
Source: Int J Drug Policy. Author manuscript; Available in PMC 2025 Oct 10. (PMC12513397; doi:10.1016/j.drugpo.2023.104156)
Supplement: supplement [file NIHMS2112334-supplement-supplement.docx]

Supplement

Supplemental Table 1: Inclusion criteria defining cannabis industry affiliated organizations and individuals

| Affiliate | Code | Inclusion criteria |
| --- | --- | --- |
| Cannabis Business | 1 | Organization is listed as having a Marijuana Business License with the Colorado Marijuana Enforcement Division or another state licensing entity |
| Cannabis Affiliated Organization | 2 | Organization shares board members, owners, or notable investors with a cannabis business |
| Cannabis Trade Association | 3 | Organization has members who are cannabis businesses |
| Cannabis Adjacent Organization | 4 | Organization or individual directly profits from the growth of the cannabis industry (cannabis agricultural supplies, consulting, technology, real estate, investment, etc.) |
| Cannabis employee | 5 | Individual is employed by or owns a cannabis business |
| Cannabis Consultant | 6 | Individual is employed by a consultancy with cannabis business clients |
| Cannabis Consultancy | 7 | Organization represents cannabis businesses in a lobbying or PR capacity |
| Hemp Company/employee | 8 | Hemp/CBD Company or affiliated individual |

Supplemental Table 2: Recipient committee types

| Committee Type | Code | Description |
| --- | --- | --- |
| Issue Committee | 1 | Any person, other than a natural person, or any group of two or more persons, including natural persons, that has: A major purpose of supporting or opposing any ballot issue or ballot question; and accepted or made contributions or expenditures in excess of $200 to support or oppose any ballot issue or ballot question or; printed two hundred or more petition sections.^1^ |
| Small Scale Issue Committee | 2 | An issue committee that does not accept contributions or make expenditures exceeding $5,000 during an election cycle for the major purpose of supporting or opposing a ballot issue or ballot question.^1^ |
| Recall Committee | 3 | An issue committee formed to support or oppose the recall of a public officer.^1^ |
| 527 Political Organization | 4 | A Federal Elections Commission (FEC) regulated party, committee, association, fund or other organization (whether or not incorporated), organized and operated primarily for the purpose of accepting contributions or making expenditures, or both, for an exempt function. ^2^ |
| Federal Political Action Committee | 5 | An FEC regulated separate segregated fund, nonconnected committee, Super PAC, Hybrid PAC or Leadership PAC.^3^ |
| Political Committee (PC) | 6 | Any person, other than a natural person, or any group of two or more persons, including natural persons that have accepted or made contributions or expenditures in excess of $200 to support or oppose the nomination or election of one or more candidates.^1^ |
| Small Donor Committee (SDC) | 7 | A form of political committee that may only accept contributions of $50 or less per person, per calendar year from natural persons who are U.S. citizens.^1^ |
| Independent Expenditures Committee (IEC) | 8 | One or more persons that makes an independent expenditures in excess of $1,000, in aggregate, or that collects in excess of $1,000 from one or more persons for the purpose of making an independent expenditure.^1^ |
| Political Party Committee | 9 | Any group of registered electors who, by petition or assembly, nominate candidates for the official general election ballot.^1^ |
| Candidate Committee | 10 | A committee making contributions and expenditures under the authority of a candidate.^1^ |
| Candidate Only (Standalone) | 11 | Candidates without a candidate committee.^1^ |

Sources: Colorado Secretary of State Campaign Finance Manual,^1^ the Federal Elections Commission website,^3^ and the Internal Revenue Service Website.^2^

Supplemental Table 3: Top 15 Cannabis industry affiliated contributors 2005-2021

| Contributor | Contributor type | Amount |
| --- | --- | --- |
| Marijuana Policy Project | Non-profit advocacy | $1,679,073 |
| Beyond Broadway | Business | $274,072 |
| Safer Voter Education Fund | Non-profit advocacy | $236,808 |
| Marijuana Policy Project Foundation | Non-profit advocacy | $123,369 |
| Christopher Woods | Individual: Owner of Terrapin Care Station and former MPP board member | $89,865 |
| Marijuana Industry Group | Trade association | $69,965 |
| The Green Solution | Business | $64,853 |
| Mary Kay Hogan | Individual: Fulcrum Group lobbyist for Good Chemistry | $54,564 |
| Kara Miller | Individual: Gold Dome Access lobbyist for the Marijuana Industry Group | $46,559 |
| Colorado Leads | Trade association | $44,229 |
| Native Roots Parent Company | Business | $36,524 |
| Christian Sederberg | Individual: Partner at Vicente Sederberg LLP and Principal at VS Strategies, Co-founder of Simplifya and VS Tech Ventures | $35,357 |
| Robert Lucero | Individual: Owner of The Spot 420 | $33,910 |
| Bruce Nassau | Individual: Marijuana Industry Group board chair 2019 and owner of Lit dispensary | $32,206 |
| Landon Gates | Individual: Capitol Focus lobbyist for the Marijuana Industry Group | $31,787 |

Supplemental Table 4: Top 15 recipients of cannabis industry funds 2005-2021

| Recipient | Committee type | Amount |
| --- | --- | --- |
| CAMPAIGN TO REGULATE MARIJUANA LIKE ALCOHOL | Issue Committee | $1,375,423 |
| COALITION TO END MARIJUANA PROHIBITION | Issue Committee | $531,593 |
| GROWING PUEBLO'S FUTURE | Issue Committee | $402,362 |
| COLORADO HEALTH RESEARCH COUNCIL | Issue Committee | $330,515 |
| ALCOHOL-MARIJUANA EQUALIZATION INITIATIVE COMMITTEE | Issue Committee | $210,768 |
| BOLD COLORADO | Indep. Expenditures Committee | $199,367 |
| SENATE MAJORITY FUND | 527 Political Organization | $135,268 |
| COLORADO DEMOCRATIC PARTY | Political Party Committee | $128,121 |
| COMMITTEE FOR RESPONSIBLE REGULATION | Issue Committee | $59,333 |
| CANNABIS COMMUNITY FOR FAIRNESS AND SAFETY | Issue Committee | $56,268 |
| SINGER FOR BOULDER COUNTY | Candidate Committee | $56,117 |
| LEADING COLORADO FORWARD | Indep. Expenditures Committee | $50,566 |
| BETTER COLORADO ALLIANCE | Indep. Expenditures Committee | $45,227 |
| PHIL WEISER FOR COLORADO | Candidate Committee | $35,960 |
| COLORADANS FOR FAIRNESS | 527 Political Organization | $32,956 |

References

1. Colorado Secretary of State. Campaign and Political Finance Manual. In:2021. <https://www.sos.state.co.us/pubs/elections/CampaignFinance/files/CPFManual.pdf>.

2. Internal Revenue Service. I. IRC 527 - POLITICAL ORGANIZATIONS. In:n.d. <https://www.irs.gov/charities-non-profits/political-organizations/exemption-requirements-political-organizations>.

3. Federal Elections Commision. Political Action Committees (PACs). <https://www.fec.gov/press/resources-journalists/political-action-committees-pacs/>. Published 2022. Accessed May 26, 2022.
